# Taxonomic revision of the genus *Machleida* Fåhraeus, 1870 (Tenebrionidae, Pimeliinae, Asidini)

**DOI:** 10.3897/zookeys.898.46465

**Published:** 2019-12-10

**Authors:** Marcin J. Kamiński, Kojun Kanda, Aaron D. Smith

**Affiliations:** 1 Northern Arizona University, Department of Biological Sciences, 617 S. Beaver St., Flagstaff, AZ 86011-5640, USA Northern Arizona University Flagstaff United States of America; 2 Purdue University, Department of Entomology, 901 W. State Street, West Lafayette, IN 47907, USA Purdue University West Lafayette United States of America; 3 Zoological Museum, Museum and Institute of Zoology, Polish Academy of Sciences, Wilcza 64, 00-679 Warsaw, Poland Zoological Museum, Museum and Institute of Zoology Warsaw Poland

**Keywords:** *
Afrasida
*, darkling beetles, female terminalia, new species, *
Scotinesthes
*, synonyms, South Afric

## Abstract

The taxonomic concept of the genus *Machleida* Fåhraeus, 1870 is tested and revised based on newly identified material. The following new species are described: *Machleida banachi*, *M. flagstaffensis*, *M. tarskii*, and *M. zofiae* Kamiński. *Machleida capillosa* Wilke, 1925 is considered as a junior subjective synonym of *Asida devia* Péringuey, 1899. *Asida lecta* Péringuey, 1899 (= *Pseudomachla recurva* Wilke, 1925) (transferred to *Afrasida*), *Machleida nossibiana* Fairmaire, 1897 (transferred to *Scotinesthes*), and *Machleida tuberosa* Wilke, 1925 (interpreted as *incertae sedis* in Asidini) are excluded from *Machleida*. An identification key for the species of the newly revised *Machleida* is provided. The present paper brings the total number of species within the genus to six (*M. banachi***sp. nov.**; *M. devia* (Péringuey, 1899); *M. flagstaffensis***sp. nov.**; *M. nodulosa* Fåhraeus, 1870; *M. tarskii***sp. nov.**; *M. zofiae* Kamiński **sp. nov.**). The morphology of female terminalia (ovipositor and genital tubes) is described for the genus for the first time.

## Introduction

Darkling beetles (Coleoptera, Tenebrionidae) are a morphologically diverse group of over 20,000 described species (Ślipiński et al. 2011), with many still being discovered every year (e.g., Aloquio et al. 2019; Ando 2019; Giraldo-Mendoza and Flores 2019; Merkl 2019; Masumoto et al. 2019; Nabozhenko and Purchart 2019; Schawaller and Bouchard 2019; Silvestro 2019; Smith and Rincon 2019; Soldati et al. 2019; Zúñiga-Reinoso et al. 2019). This publication focuses on the representatives of the subfamily Pimeliinae Latreille, 1802, specifically on *Machleida* Fåhraeus, 1870, which is one of the seven southern African genera representing Asidini Fleming, 1821 (Koch 1962).

Prior to this publication the genus *Machleida* included the following five species (Koch 1962): *M. capillosa* Wilke, 1925, *M. devia* (Péringuey, 1899), *M. lecta* Péringuey, 1899, *M. nodulosa* Fåhraeus, 1870 (type species), and *M. nossibiana* Fairmaire, 1897. All of them, except the Malgascan *M. nossibiana*, were exclusively known from single localities in the Eastern Cape Province of South Africa (Fåhraeus 1870; Péringuey 1899; Wilke 1925).

According to the most recent hypothesis, this genus can be distinguished from the other Asidini by the following combination of characters (Koch 1962): pronotum strongly cordiform, often with subangular sides; pronotal base as broad as the elytral base (except for *M. nossibiana* with narrowed pronotal base); pronotal disc with large primary sculpturing consisting at least of two median carinae on the basal half of the middle section; hypomeron with shallow, posteriorly-abbreviated antennal sulcus; elytra with large tubercles; sides of the elytral surface limited by the apically abbreviate costa; epipleuron ventral in position (not visible from above); underside of the basal segment of the protarsi simple, non-tomentose; basal segment of posterior tarsi not elongated; and outer margin of anterior tibiae inermous.

As a result of the recent efforts aimed at revising South African Asidini many previously unstudied specimens of *Machleida* were gathered. This, together with an examination of existing type material, enabled a detailed revaluation of the taxonomic status of this genus and its species components.

## Material and methods

This study was based on material from the Museum für Naturkunde der Humboldt Universität, Berlin, Germany (referred as Berlin Mus.); Natural History Museum, London, United Kingdom (British Mus.); Muséum national d’Histoire naturelle, Paris, France (Paris Mus.); Hungarian Natural History Museum, Budapest, Hungary (Budapest Mus.); and Ditsong National Museum of Natural History, Pretoria, South Africa (Transvaal Mus.). The original label data for the specimens is given in quotation marks and separated by a comma. Each line of the original label data is separated by a forward slash.

Morphological terminology mostly follows that of Doyen (1994) and Smith (2013), with additional specialized terms used for the male and female terminalia (Tschinkel and Doyen 1980; Pérez Vera 2014; Giraldo-Mendoza and Flores 2019). Terminalia were investigated using standard methodologies (see Iwan and Kamiński 2016). Morphological measurements were recorded with a filar micrometer. Length was measured along the midline from the anterior margin of the clypeus to the apex of the elytra. Width was measured across the widest points.

The phylogenetic species concept of Wheeler and Platnick (2000) is employed, as in several recent taxonomic works (Smith et al. 2011; Smith 2013; Smith and Wirth 2016). This species concept is appropriate due to its emphasis on character transformations between species and the lack of available data beyond adult morphology and distribution for any of the species in the genus.

Images were taken using a Canon 1000D body with accordion bellows and a Canon EF 100 mm macro lens. Drawings were prepared in Photoshop CS5 using photographs as templates. The distribution of species was illustrated using Quantum GIS (QGIS) v. 2.4, while the vector layers were downloaded from the Natural Earth web-page (http://www.naturalearthdata.com). The division of the Afrotropical Realm into ecoregions follows Olson et al. (2001). The list of all investigated localities is presented in Appendix 1.

## Taxonomy

### Genus Machleida

Taxon classificationAnimaliaColeopteraTenebrionidae

Fåhraeus, 1870

D03756F1-21D9-5C3A-A874-E88943BD80BE

Machleida Fåhraeus, 1870: 256
= Machloida Rye, 1873: 286. Type species: Machleida nodulosa Fåhraeus, 1870, by monotypy. Note. Unjustified emendation of Machleida Fåhraeus, 1870, not in prevailing usage.

#### Type species.

*Machleida nodulosa* Fåhraeus, 1870; by monotypy.

#### Revised diagnosis.

Exclusion of *Asida lecta* Peringuey, 1899, *Machleida nossibiana* Fairmaire, 1897, and *Machleida tuberosa* Wilke, 1925 (see below) from *Machleida* increased the morphological consistency of the genus. As a result, some of the previously listed diagnostic characters needed to be revised (e.g., sculpture of pronotum). Additionally, the present investigation reveals that some of the characters proposed by Koch (1962) are too variable within Afrotropical Asidini to be sustained as diagnostic for *Machleida*. A revised diagnosis is presented below.

**Figure 1.**
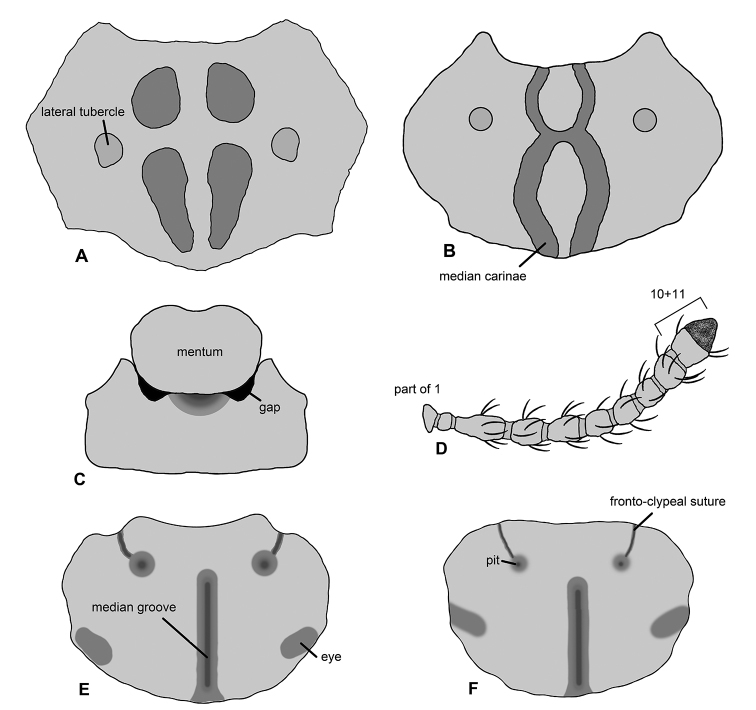
Selected diagnostic characters proposed for *Machleida* and its species. **A, B** Pronotal disc with specific surface modifications **C** ventral aspect of postoral regions **D** antenna **E, F** dorsal aspect of head. Illustrated species: **A, D***Machleida zofiae***B***M. nodulosa***C***M. tarskii***E***M. devia***F***M. flagstaffensis*.

**Figure 2.**
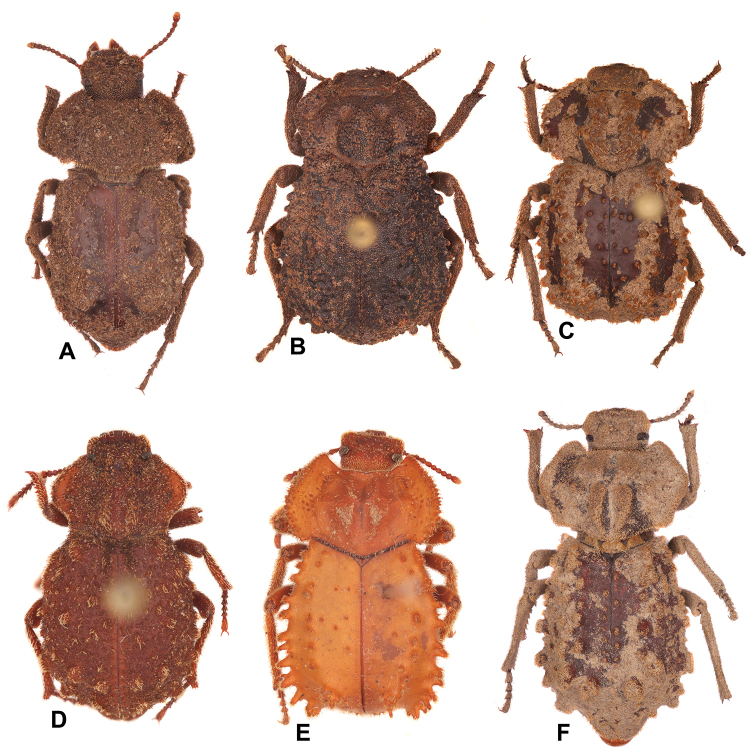
Habitus images of the studied species. **A***Machleida banachi***B***M. devia***C***M. flagstaffensis***D***M. nodulosa***E***M. tarskii***F***M. zofiae*.

**Figure 3.**
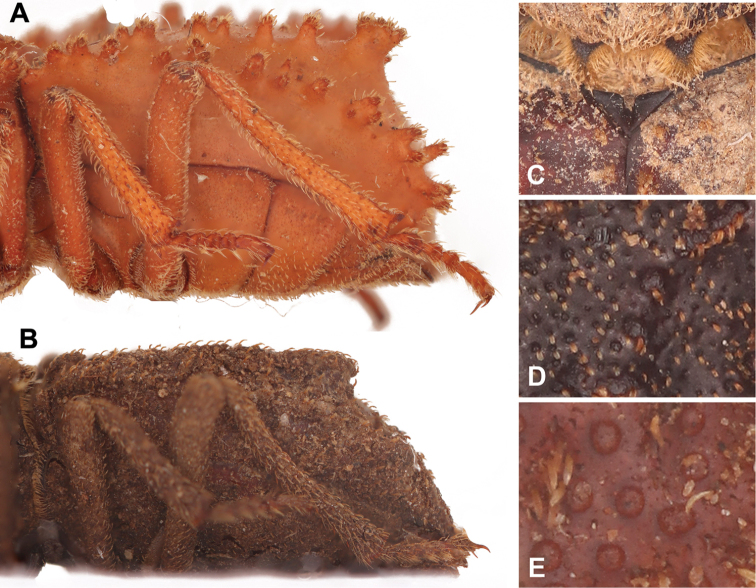
Morphological details of selected *Machleida* species. **A, B** Lateral aspect of elytron **C** scutellum and **D, E** elytral disc. Illustrated species: **A***Machleida tarskii***B***M. banachi***C***M. zofiae***D***M. devia***E***M. nodulosa*.

The representatives of this genus can be distinguished from other Afrotropical Asidini by the following combination of characters: antenna appearing as 10-segmented, with antennomeres 10 and 11 of equal in width (Fig. 1D); mentum reduced basally, not fully filling buccal cavity (Fig. 1C); pronotal disc with large primary sculpturing consisting of two median carinae merged in the middle (Fig. 1B), carinae not merging in *M. zofiae* Kamiński sp. nov. (Fig. 1A); hypomeron with shallow, posteriorly abbreviated antennal sulcus; elytra with large tubercles (Fig. 2); and expanded epipleura (sometimes fully fused with the neighbouring part of elytra) (Fig. 3A, B). Moreover, all *Machleida* species shares a peculiar structure of mesoprescutum, i.e. base deeply emarginate (Fig. 3C).

#### Species composition (6).

*M. banachi* sp. nov.; *devia* (Péringuey, 1899); *flagstaffensis***sp. nov.**; *nodulosa* Fåhraeus, 1870; *tarskii* sp. nov.; *zofiae* Kamiński sp. nov.

#### Excluded species (lecta, nossibiana, tuberosa).

**Figure S1.**
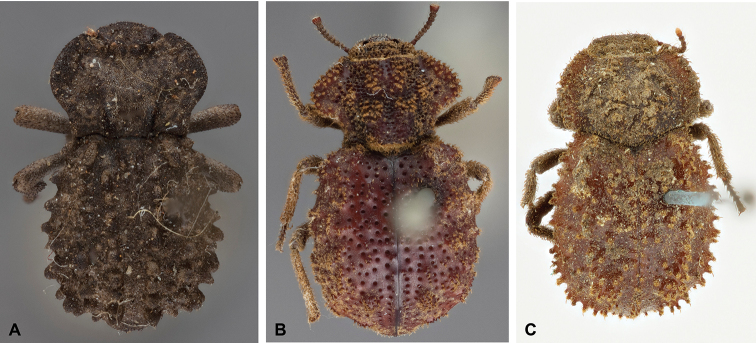
Habitus photographs of the species hereby excluded from *Machleida*. **A** Syntype of *Pseudomachla recurva* Wilke, 1925 synonym of Afrasida (Archasida) lecta (Peringuey, 1899) comb. nov. **B** holotype of *Scotinesthes nossibianus* (Fairmaire, 1897) comb. nov. **C** holotype of *tuberosa* Wilke, 1925 (*incertae sedis*).

These species are hereby excluded from *Machleida* based on differences in the structure of the mentum (fully filling buccal cavity), prosternal process (base straight in lateral view, process not convex), and pronotum (disc only basally with median carinae, lateral tubercles absent). *Asida lecta* Péringuey, 1899 does not fall within the newly formulated concept of *Machleida*. The aforementioned pronotal structure place this species within the subgenus Archasida Wilke 1922 of *Afrasida* Wilke, 1925 (Koch 1962). As a result, the following new combination is proposed: *Afrasida* (*Archasida) lecta* (Péringuey, 1899) comb. nov. A habitus photo of this species is presented in Appendix 2: Fig. S1A.

Because of its Malagasy distribution, the taxonomic placement of *M. nossibiana* Fairmaire, 1897 within the South African *Machleida* was previously questioned by several authors (Chatanay 1914; Wilke 1925; Gebien 1937). However, based on a single non-typical specimen, Koch (1962) tried to provide some morphological support for this taxonomic hypothesis. According to his view *M. nossibiana* generally resembles species of *Machleida* and can be separated from other Malagasy Asidini by the non-soleate underside of the tarsi. In his diagnosis he compared this species to *M. nodulosa* and highlighted two main morphological differences: antennae robust in *M. nossibiana*, slender in *M. nodulosa*; and prosternal process broad in *M. nossibiana*, narrow in *M. nodulosa*. The current reinvestigation of the type material of *M. nossibiana* (Appendix 2: Fig. S1B) revealed a high morphological resemblance of this species to representatives of the genus *Scotinesthes* Fairmaire, 1895 (Koch 1962). Namely, the aforementioned characters used by Koch to separate *M. nossibiana* from *M. nodulosa* are characteristic for *Scotinesthes*. Moreover, *M. nossibiana* shares a common structure of the mentum (fully filling buccal cavity; reduced basally in *Machleida*) with the other species representing that Malagasy genus. As a result, *nossibiana* is transferred from *Machleida* and the following new combination is introduced: *Scotinesthes nossibianus* (Fairmaire, 1897) comb. nov.

Reinvestigation of the type material revealed that *Machleida tuberosa* Wilke, 1925 has a peculiar pronotal sculpturing, i.e., disc without carinae but densely covered with small setose tubercles (Appendix 2: Fig. S1C). The second character seems to be unique for this species among the other southern African Asidini. Because of the aforementioned features, *M. tuberosa* does not fit the newly proposed taxonomic concept of *Machleida* and is hereby excluded from this genus. The exact placement of this species (possibly a new genus) requires further investigation in a wider taxonomic context. At the moment *tuberosa* is treated as *incertae sedis*Asidini.

#### Distribution.

Representatives of this genus have been collected in the following ecoregions of South Africa (Fig. 5): Drakensberg Montane Woodlands and Grasslands, KwaZulu-Cape coastal forest mosaic, Maputaland-Pondoland bushland, and thickets, Southern Africa mangroves.

### Key to the species of the genus *Machleida*

**Table:** 

1	Pronotal disc with two median carinae merging in middle of pronotum (Fig. 1B); lateral tubercles situated above half pronotal length (in some cases tubercles merged with median carinae) (Fig. 1B)	**2**
–	Pronotal disc with median carinae not merging in of middle of pronotum (Fig. 1A); lateral tubercles situated below half pronotal length (Fig. 1A)	***Machleida zofiae* Kamiński sp. nov.**
2	Body size = 13.0–15.0 mm	**3**
–	Body size = 7.0–9.5 mm	**4**
3	Lateral sides of pronotum sinuate (Fig. 2B). Elytral disc rugose and densely covered with microtubercles (Fig. 3D)	***Machleida devia***
–	Lateral sides of pronotum rounded. Elytral disc sparsely covered with tubercles; surface between them glabrous	***Machleida flagstaffensis* sp. nov.**
4	Elytral tubercles distributed evenly, not forming ridges (Fig. 2D). Elytra densely covered with noticeable punctures (Fig. 3E)	***Machleida nodulosa***
–	Elytral tubercles absent or sparse in middle of disc, laterally forming at least two lateral ridges (Fig. 2E). Elytra impunctate or with extremely sparse punctures	**5**
5	Elytral disc only basally with pair of oblong tubercles (middle part of elytra without tubercles). Elytral humerus dentate, protruding laterad (Fig. 2A). Elytral slope gradually falling towards apex of elytra (Fig. 3B)	***Machleida banachi* sp. nov.**
–	Median part of elytral disc covered with tubercles. Elytral humerus obtuse, not protruding laterad (Fig. 2E). Elytral slope extremely steep (Fig. 3A)	***Machleida tarskii* sp. nov.**

### Redescriptions

#### Machleida devia

Taxon classificationAnimaliaColeopteraTenebrionidae

(Péringuey, 1899)

71528F9C-04E2-5B66-98BA-B8ACFE354A77

**Figure 4.**
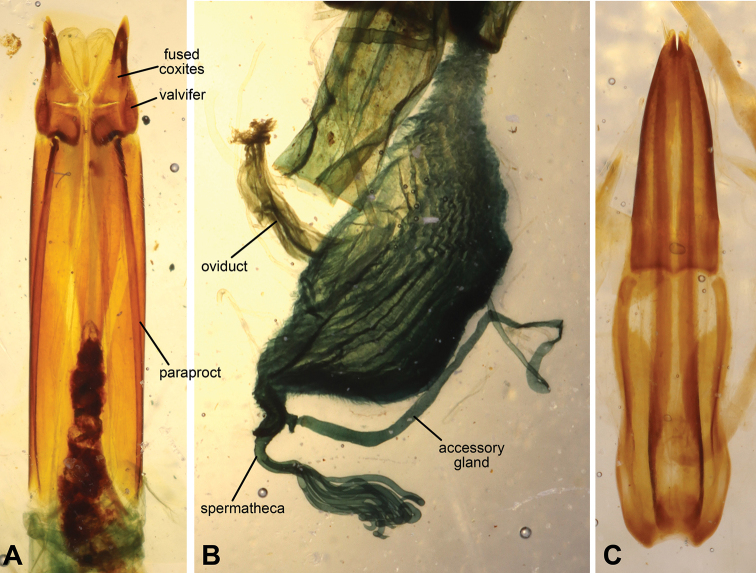
Female and male terminalia of *Machleida*. **A, B** Ovipositor and genital tubes of *Machleida flagstaffensis***C** aedeagus of *M. devia*.

**Figure 5.**
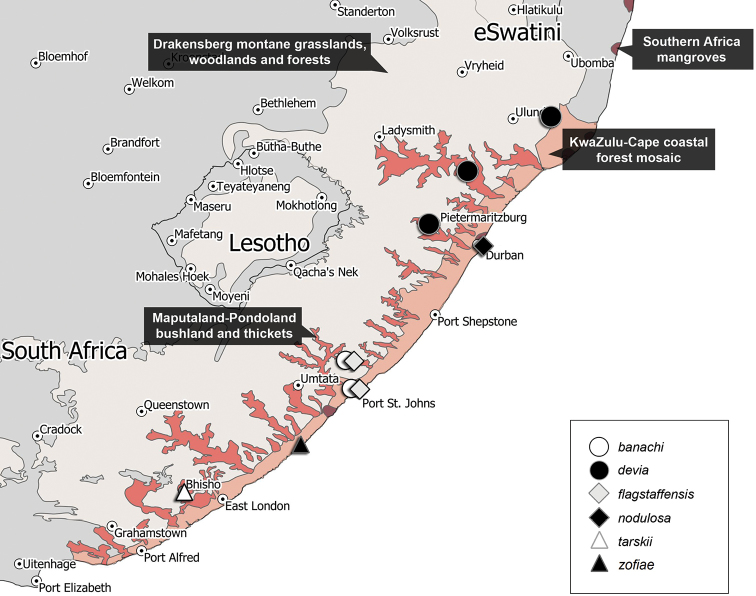
Distribution of species of the genus *Machleida*.

Figs 1E , 2B , 3D , 4C , 5

Asida devia Péringuey, 1899: 258 [transferred to Machleida by Wilke (1925: 536)]
= Machleida capillosa Wilke, 1925: 536 [syn. nov.]

##### Material studied.

***Syntype*** of *Machleida capillosa* (Berlin Mus.): “Natal Mus., / Maritzburg. / 1913-330”, “capillosa / sp. n.”. Two specimens (Transvaal Mus.): Tugela River / nr Kranskop / Lawrence & / Haacke; single specimen (Transvaal Mus.): “S. Afr.: Zululand / Hluhluwe Game Res. / 28.05S–32.04E”, “20.111992: E-Y: 2840 / fung. Trunk & litter / leg. Endrody – Younga”.

##### Notes.

During the preparation phase for this study the holotype of *Asida devia* was not found in any of the queried collections, i.e. Iziko Museum of South Africa in Cape Town and the institutions listed in the Material and methods section. However, the original description indicates several unique morphological features of this species (i.e. large body size, presence of densely distributed microtubercles on the elytral disc), which were used to differentiate it from other congeners.

##### Redescription.

Length 13.0–14.0 mm, width of elytra 8.5–9.5 mm. Integument black, often densely coated with debris. ***Head***: frons with longitudinal median depression, densely punctate (~0.2 diameters apart), each puncture with short yellowish rectangular, flattened scale-like seta; frontoclypeal suture medially indistinguishable, weakly indented at margins, with pair of lateral depressions; apical clypeal margin broadly and shallowly emarginate; clypeus projected toward front of body (Fig. 1E); apical margin of labrum strongly and sharply emarginate, densely punctate (~0.2 diameters apart), each puncture with short yellowish acuminate seta. Eye elongate oval, length approximately 8× width, weakly emarginate around epistomal base. Mentum with rounded base, not fully filling buccal cavity; anterior margin weakly medially emarginate; densely punctate, each puncture with single rectangular flattened scale-like seta. Submentum semicircular, concave medially, densely punctate. Antenna moderately clothed in erect acuminate yellowish setae; length of antennomeres 10+11 equal to 0.8 of antennomere 3 length; length of antenna equal to 0.75 of pronotal length. ***Prothorax***: pronotal lateral margin sinuate, slightly raised. Pronotum widest below middle. Disc with two median carinae merging in middle; lateral tubercles confluent with median carinae, forming convexities situated above half pronotal length; surface densely punctate (~0.2 diameters apart), each puncture with short yellowish rectangular, flattened scale-like seta; anterior margin strongly emarginate, anterior apices strongly produced; base bisinuate. Hypomeron with shallow antennal sulcus, densely punctate (~0.2 diameters apart), each puncture with short yellowish rectangular, flattened scale-like seta. Prosternal process strongly convex, without median sulcus (ventral view). ***Pterothorax***: scutellum without median groove. Elytra widest behind middle, clothed with extremely short yellowish rectangular, flattened scale-like setae and microtubercles (Fig. 3D); marginal costae present, tuberculate, apex of each tubercle densely covered with acuminate setae, divided near humera, with marginal branch extending to approximately middle of 4^th^ abdominal ventrite, dorsal branch extending to base of 3^rd^ abdominal ventrite, terminal tubercles transverse; disc rugose, without any trace of intervals and rows, covered with microtubercles; ventral portion of elytra basally impunctate. Elytral slope steep (falling at angle of 75°). Epipleura clearly distinguishable. Mesanepisternum, mesepimeron, and metepimeron impunctate or sparsely punctate. Meso- and metaventrite densely punctate and covered with acuminate setae. Lateral regions of metaventrite (between coxae) extremely short. ***Legs***: apex of profemora with small denticle on outer margin. Femora and tibia densely punctate and setose. Tarsi cylindrical, not flattened. ***Abdomen***: ventrites 1–3 moderately punctate and weakly rugulose; ventrites 4 and 5 densely punctate and setose; ventrite 5 without submarginal sulcus. ***Terminalia***: aedeagus bipartite, with apical part slightly shorter than basal portion (Fig. 4C). Female specimens were not dissected due to scarcity of available materials.

##### Notes on synonymy.

Type investigation of *Machleida capillosa* did not reveal any characters to support its taxonomic distinctiveness from *Asida devia* (e.g. both share a unique structure of elytral disc – surface densely covered with microtubercles) (Péringuey 1899). Furthermore, both species have the same *locus typicus* (“Maritzburg”, South Africa). As a result, M. *capillosa* is considered here as a junior subjective synonym of *Asida devia*.

##### Distribution.

Representatives of this species have been collected in the following ecoregions of South Africa (Fig. 5): Drakensberg Montane Woodlands and Grasslands, Maputaland-Pondoland bushland and thickets.

#### Machleida nodulosa

Taxon classificationAnimaliaColeopteraTenebrionidae

Fåhraeus, 1870

976A3654-BE9A-5EC1-B83C-EE027E910A40

Figs 1B , 2D , 5

Machleida nodulosa Fåhraeus, 1870: 256
= Asida legitima Péringuey, 1899: 266 [syn. by Péringuey (1904: 296)]

##### Material studied.

Single specimen (Transvaal Mus.): “Umkomaas / III.1951 / A.L. Capener”; single specimen (Berlin Mus.): “Natal / Durban”; single specimen (Transvaal Mus.): “Malvern / Natal”; single specimen (Budapest Mus.): “Natal / P. Reineck”, “Machleida / nodulosa / Fahr”; single specimen (Transvaal Mus.): “Lower / mKomas N. / 9/97”, “Durban Museum”; single specimen (Transvaal Mus.): “Tongaat / V.1953 / Mrs. R. Koch”.

##### Notes.

The last researcher to study the types of *Machleida nodulosa* and *Asida legitima* was Péringuey (1904). This fact indicates that those specimens should be preserved in the Iziko Museum of South Africa (Cape Town). However, the types of those both synonymous species were not found in any of the queried collections, i.e. Iziko Museum of South Africa in Cape Town and the institutions listed in the Material and methods section.

Descriptions of *M. nodulosa* and *A. legitima* indicate several specific morphological features (i.e. relatively small body size; presence of dense punctures on elytra; elytral tubercles distributed evenly, not forming ridges), which were used to differentiate them from other congeners.

##### Redescription.

Length 8.5–9.0 mm, width of elytra 4.5–5.0 mm. Integument brownish, often densely coated with debris. ***Head***: frons with shallow longitudinal median depression, densely punctate (~0.2 diameters apart), each puncture with short yellowish rectangular, flattened scale-like seta; frontoclypeal suture medially indistinguishable, weakly indented at margins, with pair of lateral depressions; apical clypeal margin broadly shallowly emarginate; clypeus slightly projected toward front of body; apical margin of labrum sharply emarginate medially, densely punctate in apical half (~0.2 diameters apart), each puncture with short yellowish setae. Eye elongate oval, length approximately 4× width, weakly emarginate around epistomal base. Mentum with rounded base, not fully filling buccal cavity, anterior margin weakly medially emarginate; densely punctate, each point with single acuminate seta. Submentum semicircular, slightly concave medially, densely punctate. Antenna moderately clothed in erect acuminate yellowish setae; length of antennomeres 10+11 equal to 0.8 of antennomere 3 length; length of antenna equal to 0.7 of pronotal length. ***Prothorax***: pronotal lateral margin strongly sinuate, slightly raised. Pronotum widest in middle. Disc with two median carinae merging in middle, lateral tubercles situated above half pronotal length; surface densely punctate (~0.2 diameters apart), each puncture with short yellowish rectangular, flattened scale-like seta; anterior margin strongly emarginate, anterior apices strongly produced; base v-shaped. Hypomeron with shallow antennal sulcus, sparsely punctate to impunctate, each puncture, if present, with short yellowish acuminate seta. Prosternal process strongly convex, longitudinally depressed in middle (ventral view). ***Pterothorax***: scutellum without median grove. Elytra widest behind middle, densely punctured and evenly covered with tubercles (tubercles sometimes merging), each tubercle clothed with short yellowish rectangular, flattened scale-like setae; ventral portion of elytra, except epipleuron, similarly structured as dorsal side. Elytral slope gradually falling towards elytral apex (at angle of 50°). Epipleuron clearly distinguishable. Mesanepisternum, mesepimeron, and metepimeron sparsely punctate. Meso- and metaventrite densely punctate and covered with acuminate setae. Lateral regions of metaventrite (between coxae) extremely short. ***Legs***: apex of profemora with small denticle on outer margin. Femora and tibia densely punctate and setose. Tarsi cylindrical, not flattened. ***Abdomen***: ventrites 1–3 moderately punctate and setose (small brownish setae); ventrites 4 and -5 densely punctate and setose; ventrite 5 without submarginal sulcus. ***Terminalia***: ovipositor and genital tubes as in *M. flagstaffensis* (Fig. 4AB). Male specimens were not dissected due to scarcity of available materials.

##### Distribution.

Representatives of this species have been collected in the following ecoregion of South Africa (Fig. 5): Southern Africa mangroves.

### New species

#### Machleida banachi sp. nov.

Taxon classificationAnimaliaColeopteraTenebrionidae

0D4F38E5-A104-5C18-8BFD-E0C2ED06C5FF

http://zoobank.org/1F446410-4A21-44B1-B134-56EBB90DEFB7

Figs 2A , 5

##### Type material.

***Holotype*** (Transvaal Mus.): “26.11.1988; E-Y:2582 / forest floor litter / leg. Endrody-Younga”, “S. Afr., Transkei / Ntsubane forest / 31.27S–29.44E”. ***Paratypes*** (Transvaal Mus.): single specimen: same data as holotype; single specimen: same data, except “1.12.1988; E-Y:2593 / forest floor litter / leg. Endrody-Younga”; single specimen: same data, except “25.11.1988; E-Y:2579 / forest floor litter / leg. Endrody-Younga”; two specimens: same data, except: 25.11.1988; E-Y:2580 / groundtraps, 14 days / leg. Endrody-Younga”, “groundtrap with / banana bait”; two specimens: same data, except “25.11.1988; E-Y:2537 / fungi & for. litter / leg. Endrody-Younga”; single specimen: “Z.A.82 / Port St. John D. / Ingogo Forest”, “Humus / XII-1961”, “N. Leleup leg.”.

##### Diagnosis.

This species can be distinguished from all its congeners by the unique structure of elytra: disc medially lacking tubercles and dentate humerus (strongly protruding laterad) (Fig. 2A). This species most closely resembles *Machleida tarski* (see identification key above).

##### Description.

Length 8.0–9.5 mm, width of elytra 4.0–7.0 mm. Integument brownish, often densely coated with debris. ***Head***: frons with longitudinal median depression, densely punctate (~0.2 diameters apart), each puncture with short yellowish rectangular, flattened scale-like seta; frontoclypeal suture medially indistinguishable, weakly indented at margins, with pair of lateral depressions; apical clypeal margin broadly shallowly emarginate; clypeus slightly projected toward front of body; apical margin of labrum sharply emarginate medially, densely punctate in apical half (~0.2 diameters apart), each puncture with short yellowish aciminate setae. Eye elongate oval, length approximately 5× width, weakly emarginate around epistomal base. Mentum with rounded base, not fully filling buccal cavity; anterior margin weakly medially emarginate; densely punctate, each with single acuminate seta. Submentum semicircular, concave medially, densely punctate. Antenna moderately clothed in erect acuminate yellowish setae; length of antennomeres 10+11 equal to 0.8 of antennomere 3 length; length of antenna equal to 0.75 of pronotal length. ***Prothorax***: pronotal lateral margin rounded, strongly raised. Pronotum widest below middle. Disc with two median carinae merging in middle; lateral tubercles confluent with median carinae, forming convexities situated above half pronotal length; surface densely punctate (~0.2 diameters apart), each puncture with short yellowish rectangular, flattened scale-like seta; anterior margin strongly emarginate, anterior apices strongly produced; base bisinuate. Hypomeron with shallow antennal sulcus, sparsely punctate to impunctate, each puncture, if present, with short yellowish acuminate seta. Prosternal process strongly convex, longitudinally depressed in middle (ventral view). ***Pterothorax***: scutellum without median grove. Elytra widest behind middle, clothed with short yellowish rectangular, flattened scale-like setae; marginal costae present, tuberculate, apex of each tubercle densely covered with setae, marginal branch extending to approximately apex of 4^th^ abdominal ventrite, dorsal branch extending to apex of 3^rd^ abdominal ventrite, terminal tubercles transverse; disc smooth, without any trace of intervals, sparsely covered with flattened setae, with elongated tubercles near base (Fig. 3B); ventral portion of elytra basally impunctate, apically with sparse punctures. Elytral slope gradually falling towards elytral apex (at angle of 50°). Epipleura indistinguishable from neighbouring portion of elytra. Mesanepisternum, mesepimeron, and metepimeron impunctate or sparsely punctate. Meso- and metaventrite densely punctate and covered with setae. Lateral regions of metaventrite (between coxae) extremely short. ***Legs***: Apex of profemora with small denticle on outer margin. Femora and tibia densely punctate and setose. Tarsi cylindrical, not flattened. ***Abdomen***: ventrites 1–3 moderately punctate and weakly rugulose; ventrites 4 and 5 densely punctate and setose; ventrite 5 without submarginal sulcus. ***Terminalia***: aedeagus as in *M. devia* (Fig. 4C). Female specimens were not dissected due to scarcity of available materials.

##### Etymology.

This newly introduced name honours Stefan Banach (30 March 1892–31 August 1945), prominent Polish mathematician and founder of modern functional analysis. He was educated at the Technical University of Lwów and was a founder of the Lwów School of Mathematics.

##### Distribution.

Representatives of this species have been collected in the following ecoregions of South Africa (Fig. 5): KwaZulu-Cape coastal forest mosaic, Maputaland-Pondoland bushland and thickets.

#### Machleida flagstaffensis sp. nov.

Taxon classificationAnimaliaColeopteraTenebrionidae

5E99ED5A-61F2-5EA7-A602-9E679C266931

http://zoobank.org/1D55CB36-328A-4CA7-BA51-27434AEF42EF

Figs 1F , 2C , 4A, B , 5

##### Type material.

***Holotype*** (Transvaal Mus.): “1.12.1988; E-Y:2593 / forest floor litter / leg. Endrody-Younga”, “S. Afr., Transkei / Ntsubane forest / 31.27S–29.44E”. ***Paratypes***: single specimen (Transvaal Mus.): same data as holotype; single specimen (Transvaal Mus.): “24.11.1987; E-Y:2533 / indig. Forest litter / leg. Endrody-Younga”, “S. Afr., Transkei / Silaka For. Reserve / 31.33S–29.30E”

##### Diagnosis.

On account of a large body size this species is similar to *Machleida devia*. Both species can be separated by the characters listed in the identification key provided above.

##### Description.

Length 13.0–15.0 mm, width of elytra 7.0–7.5 mm. Integument brownish, often densely coated with debris. ***Head***: frons with longitudinal median depression, densely punctate (~0.2 diameters apart), each puncture with short yellowish acuminate seta; frontoclypeal suture medially indistinguishable, weakly indented at margins, with pair of lateral depressions; apical clypeal margin broadly shallowly emarginate; clypeus projected toward front of body (Fig. 1F); apical margin of labrum sharply emarginate, densely punctate (~0.2 diameters apart), each puncture with short yellowish seta. Eye elongate oval, length approximately 6× width, weakly emarginate around epistomal base. Mentum with rounded base, not fully filling buccal cavity, anterior margin weakly medially emarginate; densely punctate, punctures moderately sized, each with single slender setae. Submentum triangular, concave, densely punctate. Antenna moderately clothed in erect acuminate clear to yellowish setae; length of antennomeres 10+11 equal to 0.9 of antennomere 3 length; antennomeres 10+11 noticeably widended; length of antenna equal to 0.75 of pronotal length. ***Prothorax***: pronotal lateral margin rounded, slightly elevated. Pronotum widest in middle base. Disc with two median carinae merging in middle, and lateral tubercles situated above half pronotal length; surface sparsely punctate, covered with noticeable microsculpture and extremely short, brownish setae. Median carinae and remaining tubercles apically covered with elongated scale-like setae and short elongated setae; anterior margin emarginate, densely setose; base bisinuate. Hypomeron with relatively deep antennal sulcus, impunctate. Prosternal process strongly convex, rounded at apex. ***Pterothorax***: scutellum with median longitudinal grove. Elytra widest behind middle; surface between tubercles glabrous, covered with microsculpture; marginal costae present, tuberculate, apex of each tubercle densely covered with numerous short acuminate setae and large single scale-like seta apically, divided near humera, marginal branch extending to approximately middle of 4^th^ abdominal ventrite, dorsal branch extending to base of 3^rd^ abdominal ventrite, terminal tubercles transverse; disc without any trace of intervals or rows, sparsely covered with tubercles; ventral portion of elytra basally impunctate. Elytral slope steep (falling at angle of 75°). Epipleuron indistinguishable from neighbouring portion of elytra. Mesanepisternum, mesepimeron, and metepimeron sparsely punctate. Meso- and metaventrite sparsely punctate and covered with setae. Lateral regions of metaventrite (between coxae) extremely short. ***Legs***: apex of profemora with small denticle on outer margin. Femora and tibia densely punctate and setose. Tarsi cylindrical, not flattened. ***Abdomen***: ventrites 1–3 sparsely punctate and setose; ventrites 4 and 5 moderately punctate and setose; ventrite 5 without submarginal sulcus. ***Terminalia***: ovipositor with paraproct much longer that coxites, plates 2–4 fused (Fig. 4A). Genital tubes similarly structured to other Asidini (Fig. 4B). Male specimens were not dissected due to scarcity of available materials.

##### Etymology.

The species epithet refers to Flagstaff (Eastern Cape, South Africa), a town near the collecting localities of this new species.

##### Distribution.

Representatives of this species have been collected in the following ecoregions of South Africa (Fig. 5): KwaZulu-Cape coastal forest mosaic, Maputaland-Pondoland bushland and thickets.

#### Machleida tarskii sp. nov.

Taxon classificationAnimaliaColeopteraTenebrionidae

FB3B8089-F701-5C94-A679-23AFB92324AE

http://zoobank.org/52AF9427-4126-49E2-BF5C-DC6BEEEA7ED2

Figs 1C , 2E , 5

##### Type material.

***Holotype*** (Transvaal Mus.): “Z.A. 86 / Pirie Forest / King Williams Town”, “Humus / XII.1961”, “N. Leleup leg.”. ***Paratype***: single specimen same data as holotype.

##### Diagnosis.

This species most closely resembles *Machleida banachi*, as both share a similar body size, structure of pronotal disc (median carinae merging) and elytra (tubercles forming two lateral ridges). They can be separated by different formation of the elytral slope (extremely steep in *tarskii*; gradually falling in *banachi*) (Fig. 3A, B). For other characters separating those both species see the identification key.

##### Description.

Length 9.0–9.2 mm, width of elytra 5.0–5.2 mm. Integument dark brown (yellowish in holotype), often densely coated with debris. ***Head***: frons with longitudinal median depression, sparsely punctate (3.0–4.0 diameters apart), each puncture with short yellowish acuminate setae; frontoclypeal suture medially indistinguishable, indented at margins, with pair of lateral depressions; apical clypeal margin broadly shallowly emarginate; clypeus slightly projected toward front of body; apical margin of labrum strongly emarginate, densely punctate apically (~0.2 diameters apart), each puncture with short yellowish setae. Eye elongate oval, length approximately 4× width, weakly emarginate around epistomal base. Mentum with rounded base, not fully filling buccal cavity, anterior margin medially emarginate; sparsely punctate, each puncture with single seta. Submentum semicircular, concave, impunctate. Antenna moderately clothed in erect acuminate clear to yellowish setae; length of antennomeres 10+11 equal that of antennomere 3; length of antenna equal to 0.75 of pronotal length. ***Prothorax***: pronotal lateral margin rounded, strongly raised, densely covered with tubercles, each tubercle densely covered with patch of short setae and medially with few scale-like longer setae. Pronotum widest below base. Disc with two median carinae merging in middle; lateral tubercles confluent with median carinae, forming convexities situated above half pronotal length; surface moderately punctate (1.0–2.0 diameters apart), punctures without setae; anterior margin strongly emarginate, anterior apices strongly produced; base bisinuate. Hypomeron with shallow antennal sulcus, impunctate. Prosternal process strongly convex, densely covered with short scale-like setae, longitudinally depressed in middle (ventral view). ***Pterothorax***: scutellum with median longitudinal grove. Elytra widest behind middle, impunctate; two marginal rows of tubercles present, apex of each tubercle densely covered with setae; marginal branch extending to approximately middle of 5^th^ abdominal ventrite, dorsal branch extending to base of 5^th^ abdominal ventrite, terminal tubercles transverse; disc without any trace of intervals, between tubercles glabrous, tubercles distributed sparsely, each apically with dense patch of setae; ventral portion of elytra, mesanepisternum, mesepimeron, and metepimeron impunctate, and sparsely covered with short, brownish setae. Elytral slope extremely steep (falling at angle of 80°). Epipleura indistinguishable from neighbouring portion of elytra. Meso- and metaventrite moderately punctate and covered with yellowish setae. Lateral regions of metaventrite (between coxae) extremely short. ***Legs***: apex of profemora with small denticle on outer margin. Femora and tibia densely punctate and setose. Tarsi cylindrical, not flattened. ***Abdomen***: ventrites 1–4 sparsely covered with brownish short setae; ventrite 5 moderately punctate and setose, (yellowish setae) without submarginal sulcus. ***Terminalia***: specimens were not dissected due to scarcity of available materials.

##### Etymology.

The species epithet is in honor of Alfred Tarski (14 January 1901–26 October 1983), Polish-American logician and mathematician. Educated at the University of Warsaw and a member of the Lwów–Warsaw school of logic, he immigrated in 1939 to the USA, where he became a citizen in 1945. Alfred Tarski carried out research in mathematics at the University of California, Berkeley, from 1942 until his death in 1983.

##### Distribution.

Representatives of this species have been collected in the following ecoregion of South Africa (Fig. 5): Drakensberg Montane Woodlands and Grasslands.

#### Machleida zofiae

Taxon classificationAnimaliaColeopteraTenebrionidae

Kamiński sp. nov.

BE40D4F5-6390-5B10-B239-0B238CE7F7FB

http://zoobank.org/721AF25A-EC87-4DD1-B138-F020C37759D1

##### Type material.

***Holotype*** (Transvaal Mus.): “Transkei: coast / Dwesa for. Res. / 32.17S–28.50E”, “26.2.1985; E-Y: 2165 / groundtraps, 7 days / leg. Endrody-Younga”, “groundtrap with / banana bait”.

##### Diagnosis.

Easily distinguishable from other congeners by the specific structure of pronotum: disc with median carinae interrupted in the middle of pronotal disc (Fig. 1A); lateral tubercles situated below the half pronotal length (Fig. 1A). This is also the only *Machleida* species with relatively large tuberculate horns on frons. Superficially this species can be confused with *M. flagstaffensis*. However, besides the characters listed above those two species can be distinguished by different structure of elytral slope, i.e. steep in *flagstaffensis* (falling at angle of 75°) versus gradually falling in *zofiae* (at angle of 50°).

##### Description.

Length 11.5 mm, width of elytra 5.5 mm. Integument brownish, densely coated with debris. ***Head***: frons with pair of tuberculate horns, densely punctate (~0.2 diameters apart), each puncture with short yellowish acuminate seta; frontoclypeal suture medially indistinguishable, indented at margins, with pair of lateral depressions; apical clypeal margin broadly and shallowly emarginate; clypeus slightly projected toward front of body; apical margin of labrum strongly emarginate, densely punctate apically (~0.2 diameters apart), each puncture with short, yellowish seta. Eye elongate oval, length approximately 4× width, weakly emarginate around epistomal base. Mentum with rounded base, not fully filling buccal cavity, anterior margin medially emarginate; sparsely punctate, each puncture with single seta. Submentum semicircular, concave, impunctate. Antenna moderately clothed in erect acuminate clear to yellowish setae; length of antennomeres 10+11 equal that of antennomere 3 (Fig. 1D); length of antenna equal to 0.85 of pronotal length. ***Prothorax***: pronotal lateral margin sinuate, strongly raised, densely covered with setae. Pronotum widest in middle. Disc with median carinae not merging in middle (Fig. 1A); lateral tubercles located below half pronotal length; surface moderately to densely punctate (0.2–2.0 diameters apart), punctures with flattened setae; anterior margin strongly emarginate, anterior apices strongly produced; base bisinuate. Hypomeron with shallow antennal sulcus, sparsely punctate or impunctate. Prosternal process strongly convex, densely covered with short scale-like setae, longitudinally depressed in middle (ventral view). ***Pterothorax***: scutellum with median longitudinal grove (Fig. 3C). Elytra widest behind middle, impunctate; two marginal rows of tubercles present, apex of each tubercle densely covered with acuminate setae, marginal branch extending to approximately base of 4^th^ abdominal ventrite, dorsal branch extending to middle of 3^th^ abdominal ventrite; terminal tubercles of both rows enlarged; disc without any trace of intervals, covered with tubercles distributed in two rows near suture, each tubercle apically with dense patch of setae, surface of disc glabrous between tubercles; ventral portion of elytra, mesanepisternum, mesepimeron, and metepimeron impunctate, sparsely covered with short brownish setae. Elytral slope gradually falling towards elytral apex. Epipleuron clearly distinguishable. Meso- and metaventrite moderately punctate and covered with yellowish setae. Lateral regions of metaventrite extremely short. ***Legs***: apex of profemora with small denticle on outer margin. Femora and tibia densely punctate and setose. Tarsi cylindrical, not flattened. ***Abdomen***: ventrites 1–4 sparsely covered with short, brownish setae; ventrite 5 moderately punctate and setose, (yellowish setae) without submarginal sulcus. ***Terminalia***: single holotype was not dissected.

##### Etymology.

*Machleida zofiae* is named in honour of the first author's daughter, Zofia Irena Kamińska, born on November 3, 2018 (Flagstaff, USA).

##### Distribution.

The holotype of this species was collected in the following ecoregion of South Africa (Fig. 5): KwaZulu-Cape coastal forest mosaic.

## Supplementary Material

XML Treatment for Machleida

XML Treatment for Machleida devia

XML Treatment for Machleida nodulosa

XML Treatment for Machleida banachi

XML Treatment for Machleida flagstaffensis

XML Treatment for Machleida tarskii

XML Treatment for Machleida zofiae

## Appendix 1

### Analysed distributional data in CSV format

Genus, Species, Locality, Latitude, Longitude

Machleida, banachi, St John, -31.6288, 29.5369

Machleida, banachi, Ntsubane forest, -31.276506, 29.439460

Machleida, devia, Maritzburg, -29.61679, 30.39278

Machleida, devia, Hluhluwe, -28.308716, 31.877668

Machleida, devia, Kranskop, -28.967373, 30.86351

Machleida, flagstaffensis, Ntsubane forest, -31.276506, 29.439460

Machleida, flagstaffensis, Silaka Reserve, -31.652780, 29.508211

Machleida, nodulosa, Durban, -29.883333, 31.05

Machleida, nodulosa, Umkomaas, -30.2, 30.8

Machleida, nodulosa, Malwern, -29.884374, 30.919393

Machleida, tarskii, King Williams Town, -32.883333, 27.4

Machleida, zofiae, Dwesa, -32.302013, 28.821885

## Appendix 2

### Label data for analysed specimens:

Afrasida (Archasida) lecta (Peringuey, 1899) comb. nov.

*Asida lecta* Peringuey, 1899 (=*Pseudomachla recurva* Wilke, 1925)

**Material studied.** Three specimens (Transvaal Mus.): “Algoa bay / CApland / Dr. Brauns / 9.2.96”; single specimen (Transvaal Mus.): “Resolution / Albany Distr. 21–22.II.1925 / A. Walton”.

*Scotinesthes nossibianus* (Fairmaire, 1897) comb. nov.

*Machleida nossibiana* Fairmaire, 1897

**Material studied.** Holotype (Paris Mus.): “Type”, “Nossibe”, “Machla / nossibiana/ Fairm Madaga / D. *unreadable*”

*tuberosa* Wilke, 1925 (*incertae sedis*Asidini)

*Machleida tuberosa* Wilke, 1925

**Material studied.** Holotype (British Mus.): “F. Steph”, “Type”, “tuberosa / sp. nov. det. S. Wilke”, “1913:466”, “NHMUK 013903124”.
